# How relevant is the basic reproductive number computed during the coronavirus disease 2019 (COVID-19) pandemic, especially during lockdowns?

**DOI:** 10.1017/ice.2020.1376

**Published:** 2020-12-14

**Authors:** Arni S.R. Srinivasa Rao, Steven G. Krantz, Michael B. Bonsall, Thomas Kurien, Siddappa N. Byrareddy, David A. Swanson, Ramesh Bhat, Kurapati Sudhakar

**Affiliations:** 1 Medical College of Georgia, Augusta, Georgia; 2 Laboratory for Theory and Mathematical Modeling, Department of Medicine - Division of Infectious Diseases, Medical College of Georgia, Augusta University, Augusta, Georgia; 3 Department of Mathematics, Washington University, St Louis, Missouri; 4 Mathematical Ecology Research Group, Department of Zoology, University of Oxford, Oxford, United Kingdom; 5 Department of Medicine, Pondicherry Institute of Medical Sciences, Puducherry, India; 6 Department of Pharmacology and Experimental Neuroscience, University of Nebraska Medical Center, Omaha, Nebraska; 7 Department of Sociology, University of California–Riverside, Riverside, California; 8 NMIMS University, Mumbai, India; 9 (formerly with) Centers for Disease Control and Prevention, World Bank, and United States Agency for International Development

*To the Editor*—The basic reproductive number 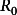 in epidemiology is defined as the average number of secondary infections that will be likely produced by a primary infected person in a predominantly susceptible population. Mathematically, it is an accurate measure of disease spread.^1^ However, the value of 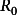 is difficult to estimate from epidemiological data, for example, during the ongoing coronavirus disease 2019 (COVID-19) pandemic. In recent studies on COVID-19, for example,^2–4^ computed a time-varying 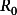 has been computed, which researchers called 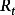. They ascertained that the decline in 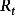 is due to continued lockdowns and nonpharmaceutical interventions. Although the conclusions in those studies are supported by the data, estimates of 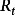 raise methodological issues that require further consideration. Here, we convey the essential and technical difficulties in estimating either 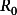 or 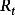 from the data, and we discuss how a model-based 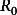 may not adequately capture the actual spread of the disease. Although these limitations are generally unavoidable (even after defining appropriate error structures and statistical modeling), the inappropriate use of this metric, especially in the ongoing COVID-19 pandemic, has important implications for infectious disease mitigation planning.

**Fig. 1.**
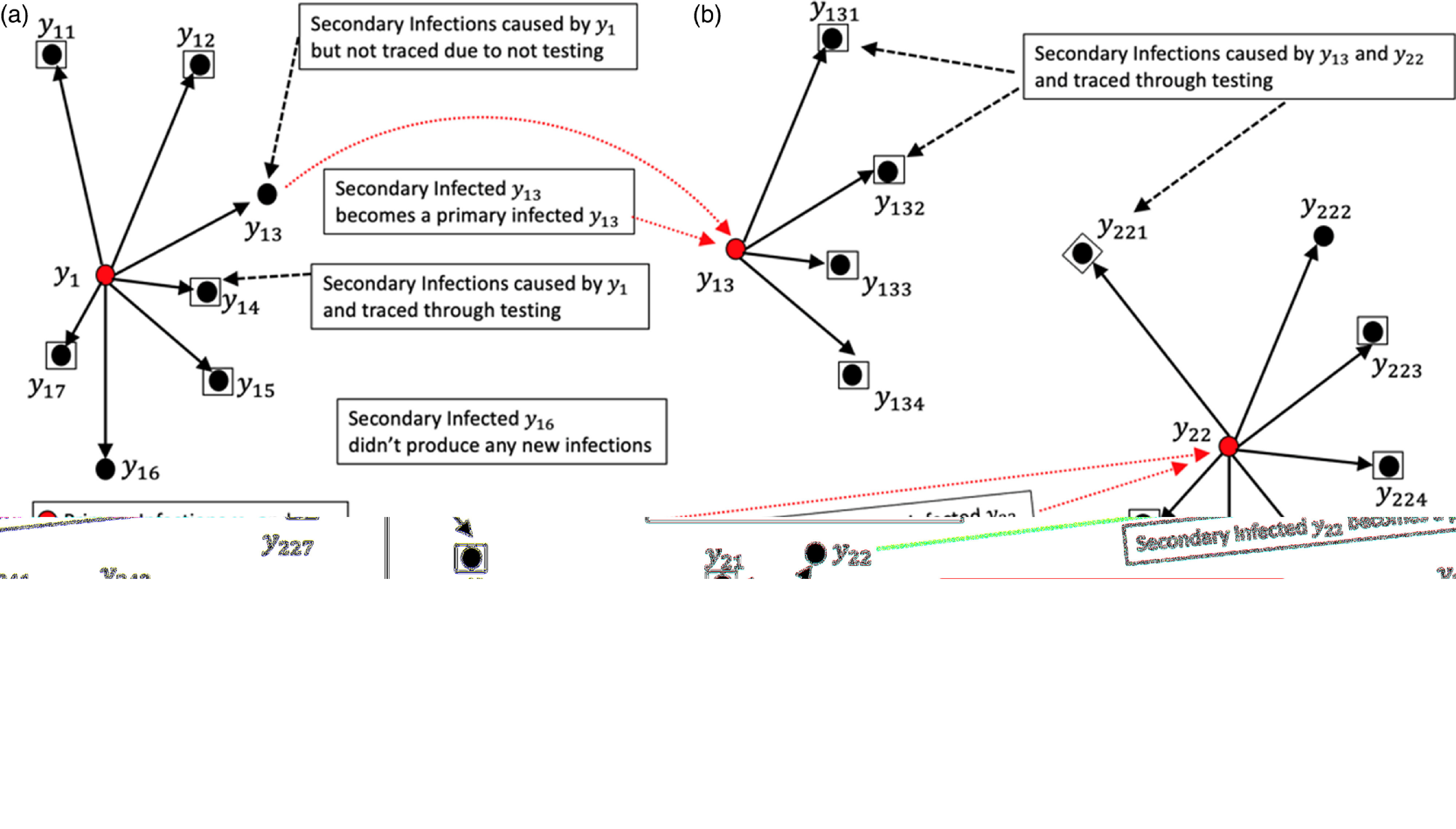
Demonstration of average number of secondary infections observed through tracing and diagnosing. In **(a)**, let 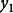 and 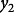 be the two primary COVID-19 infected, where the individual 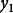 had generated 7 secondary infections out of which 5 were traced and diagnosed. The individual 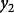 had generated 4 secondary infections out of which 2 were traced and diagnosed. The observed arithmetic average secondary infected by 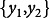 in (a) was 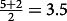, but the true average by them was 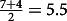. In **(b)**, the third secondary infection in **(a)**, say, 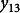 becomes a primary infected that generates 4 secondary infections out of which all were traced and diagnosed. In **(b)**, the second secondary infection in **(a)**, say, 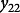 becomes a primary infected that generates 7 secondary infections out of which only 5 were traced and diagnosed. Finally, in **(b)**, the fourth secondary infection in **(a)**, say, 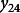 by primary infected 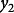 becomes a primary infected that generates 3 secondary infections out of which only 2 were traced and diagnosed. The observed arithmetic average secondary infections by 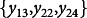 was 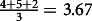, but if every COVID-19 patient was diagnosed, then the true average secondary infections by them was 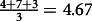. Note that the total traced and tested could be many fold more than the actual positive cases found. Suppose 22 secondary infections generated during the third generation, then the mean number of secondary infections (geometric) obtained during three generations of spread is 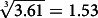.

Suppose that 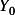 is the number of infected people at time 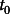 who could generate secondary infections between 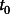 and 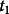, say, 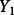. However, the testing of all the potential infected individuals during this period need not be complete. 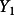 could generate further secondary infections between 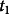 and 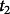, say, 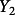, and so on. Again, the testing of the samples through contact tracing need not be complete (Fig. 1). That is, 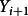 at 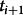 could be generated by 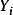 at 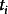 for *i* = 0, 1, …. In reality, during most epidemics, and especially for the COVID-19 pandemice, only a fraction of 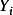, say, 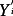 are ever reported (and also diagnosed due to incomplete testing) such that 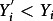 for all *i*.^5,6^ This partial reporting (including partial diagnosis and partial testing) could also be due to lockdowns and lack of proper knowledge regarding COVID-19 (forced or natural behavior changes in the community, eg, lockdowns and use of masks). The average number of secondary infections generated by 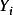 individuals is 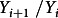. If there is variation in the infected people or a rapid aggregation of infected people, then it is more appropriate that we should use the geometric mean instead of the arithmetic mean approaches to determine expected reproductive numbers. Not only is the former far better suited than the latter to deal both with fluctuations and numbers that are not independent of one another, it also is the only correct mean when using results that are presented as ratios.^7–9^

Suppose that 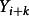 is the number of infected people at time 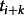 when lockdowns are introduced at *k* for *k* = 0, 1, 2 ….

Assume that(1)
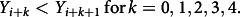



The percentage of growth in the number of infected people during the 4 time intervals (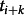, 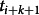) for *k* = 0, 1, 2, 3, 4, are, say, 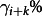 for *k* = 0, 1, 2, 3, 4, respectively. These growth percentages are computed as
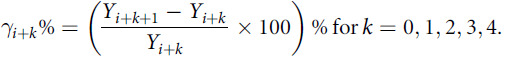



The secondary infections caused by an infected individual (Fig. 1) are the people who were not traced by the system. This step assumes that all of the infected people who were identified by the system were either quarantined or were controlled not to spread the virus further. Only a proportion of infected people who were tested and identified during lockdowns was reported, and others were either not diagnosed or not reported. Asymptomatic individuals could be anywhere in the process; that is, they were part of the identified and reported group or were among those who had not been contact traced or diagnosed. The mean (geometric) number of secondary infections would be appropriate because we were considering proportionate secondary infections. Hence, the mean number of secondary infections during (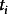, 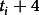) is given by(2)
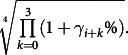



Similarly, the trend in eq. (1) continues for 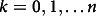, then the mean number of secondary infections during the lockdown period (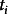, 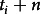) is given by(3)
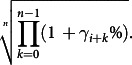



This point applies to several studies in which the reporting over time of the study is not constant. Even if the testing numbers and testing patterns are constant over a period, the proportion of underreported cases may not be constant. Thus, the estimation of 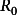 is likely to be highly variable in any given situation. For the practical purposes of computing 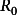 or 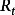 we usually have data on 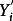, the number tested.

When the ratios 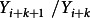 for 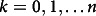 are considered, then the geometric mean of these growth rates would be(4)
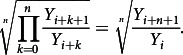



However, 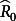 or 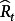, (the estimated basic and time-varying reproductive numbers at the start or ongoing through an epidemic, respectively) may not be at all close to 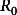 or 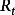 even if the 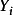 values are generated from a mathematical model for a period 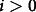 that uses data on susceptible, exposed, infected, and recovered in which the underlying epidemiological processes are time varying. This factor will introduce bias to estimates of model-based basic reproductive rates and time-varying reproductive rates. Some other limitations in various studies arise due to computing 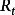 after lockdowns were relaxed. Possibly, heterogeneity exists in the data that could have masked 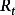 measures due to the computation of subnational and regional parameters in several COVID-19–affected countries.

The lesson here is that mathematical models must be used with care. They must be fitted to the data, and their accuracy must be carefully monitored and quantified.^10^ Any alternative course of action could lead to wrong interpretation and mismanagement of the disease with disastrous consequences.
